# Mode of delivery and offspring adiposity in late adolescence: The modifying role of maternal pre-pregnancy body size

**DOI:** 10.1371/journal.pone.0209581

**Published:** 2019-01-03

**Authors:** Maskit Bar-Meir, Yechiel Friedlander, Ronit Calderon-Margalit, Hagit Hochner

**Affiliations:** 1 Infectious Diseases, Shaare-Zedek Medical Center, Jerusalem, Israel; 2 Faculty of Medicine, Hebrew University, Jerusalem, Israel; 3 Braun School of Public Health, Hebrew University-Hadassah, Jerusalem, Israel; Mount Sinai Health System, University of Toronto, CANADA

## Abstract

**Objective:**

To study the association between mode of delivery and offspring BMI in late adolescence in a large cohort that predated the obesity epidemic, and assess the role of maternal pre-pregnancy BMI (ppBMI) in this association.

**Study design:**

We conducted a historical prospective study in the setting of the Jerusalem Perinatal Study (JPS), a population-based cohort that includes all 17,003 births to residents of West Jerusalem, between 1974 and 1976. Offspring’s BMI at age 17 was obtained upon army recruitment and was available for 11,001 of cohort participants. The associations were examined using logistic regressions, adjusting for socio-demographic characteristics and for proxies for indication for C-Section birth. Analyses were then stratified by quartiles of ppBMI.

**Results:**

C-Section was associated with offspring overweight/obesity, with adjusted OR of 1.44 (95%CI:1.14–1.82). Significant interaction of ppBMI with mode of delivery was observed, such that the associations of C-Section with overweight/obesity were limited to the upper quartile of ppBMI (adjusted OR = 1.70, 95%CI:1.18–2.43). Restricting the analyses to singleton first births and excluding pregnancies complicated with toxemia and gestational diabetes yielded similar findings.

**Conclusions:**

C-Section was positively associated with being overweight/obese at age 17. Importantly, ppBMI modified this association, with a significant association between C-Section and overweight/obesity evident only among offspring born to mothers in the highest ppBMI quartile. In light of the growing rates of obesity in women of reproductive age, these results should be considered in patient-doctor shared decisions related to selection of mode of delivery, in the absence of a clear medical indication.

## Introduction

Obesity, a global epidemic, often presents as an intergenerational cycle; parental obesity more than doubles the risk of obesity in adult offspring, and up to 80% of children with two obese parents will become obese adults [1]. Factors associated with maternal overnutrition, such as increased maternal pre-pregnancy body mass index (BMI) and gestational weight gain were shown to affect offspring adiposity [2].

In parallel, the rate of Caesarean section (C-Section) deliveries has also increased dramatically over the last decades. In 1965, C-Section comprised only 4.5% of all births in the United States, but increased to more than 30% in 2015 [3, 4].

C-Section delivery has been shown to be associated with higher risk of overweight and obesity in offspring. Two meta-analyses reported an increased risk of overweight/obesity, with pooled ORs ranging between 1.26 (95%CI:1.16,1.38) and 1.33 (95% CI: 1.19, 1.48) for offspring delivered by C-Section compared with those delivered vaginally [5, 6]. However, some of the studies included in these meta-analyses were either based on a relatively small sample size [7], did not account for major confounders such as parental obesity [8] or have followed offspring only up to late childhood. A large prospective study, following >22000 individuals from childhood through early adulthood, showed that C-Section was associated with an adjusted risk-ratio of 1.15 (95%CI: 1.06–1.26) for obesity. Moreover, individuals born by C-Section had 64% higher odds of obesity than did their siblings born via vaginal delivery [9]. There is accumulating evidence that mode of delivery plays a central role in the acquisition and structure of the initial microbiota in newborns [10], and therefore both birth mode and the infant gut microbiota may jointly contribute to offspring obesity. Indeed a recent report, based on approximately 900 mother-offspring pairs, using adiposity measured in children at ages 1 and 3 years, has demonstrated for the first time that maternal adiposity and mode of delivery together affect offspring initial microbial composition, and thereby mediate subsequent risk for early childhood obesity [11].

Here, we further investigate the association between mode of delivery and offspring adiposity, reflected by BMI, in late adolescence, and specifically assess the modifying role of maternal pre-pregnancy BMI (ppBMI) in this association. To study these questions, we make use of a large population-based birth cohort initiated during the 70’s. Identifying these associations in a cohort predating the current obesity epidemic may suggest that the underlying mechanisms are beyond the effect of changes in lifestyle associated with increased obesity risks.

## Methods

The Jerusalem Perinatal Study (JPS) population-based birth cohort includes all 17,003 births to residents of West Jerusalem, between 1974 and 1976. All pregnancies of 28 weeks or more, live births and stillbirths weighing at least 1000 grams were recorded. Detailed information on data collection has been previously described [12]. Briefly, demographic data were extracted from the hospital notification of delivery, that was sent to the district health office. This notification is mandatory by Israeli law and is complete. Demographic and clinical data were abstracted from birth certificates or maternity ward logbooks. Medical and lifestyle information, such as gestational age, mother’s smoking status, height and pre-pregnancy weight, mode of delivery and obstetric history, was collected by interviews of mothers on the first days postpartum. Data on overweight/obesity (BMI≥25kg/m^2^) in offspring at age 17 were obtained from the Israeli military draft records, available for approximately 65% of the cohort. Data on mode of delivery and adiposity at age 17 were available for a total of 11,001 births included in this study.

Logistic regressions were used to study the associations between C-Section and overweight/obesity, adjusting for socio-demographic characteristics, i.e. maternal education, socioeconomic status, ethnicity and offspring sex (Model 1), and then further controlling for proxies for indication for C-Section birth, i.e. toxemia, diabetes in pregnancy, multiple pregnancy, birth order, maternal age at delivery, smoking during pregnancy, pre- and post-term delivery (≤ 37 weeks and >41 weeks, respectively), birth weight in kg, low (≤ 2500 gr) and high (>4000 gr) birth weight and previous C-Section (Model 2). Restricted analysis on a subgroup of 9,791 singleton first births without toxemia or diabetes in pregnancy was performed as well. Analyses were then stratified by quartiles of ppBMI. The interaction between C-Section and ppBMI was tested by introducing a multiplicative term into the non-stratified models (e.g. C-Section*BW, C-Section*ppBMI). All analyses were carried out using SPSS version 21.0 statistical package (SPSS, Inc, Chicago, IL).

This study was approved by the institutional review board (IRB) of the Hadassah hospital (Hebrew university) which waived the requirement for informed consent. All data were fully anonymized.

## Results

At age 17, 12.5% of offspring were overweight/obese. Characteristics of the cohort at birth and at adolescence by mode of delivery are presented in Table 1. Overall, 7.0% of the cohort delivered through C-Section. Mothers delivering in C-Section were older and had a higher rate of toxemia and diabetes compared with mothers delivering vaginally. Additionally, vaginally delivered babies had a higher birth weight (BW) compared with C-Section delivered babies.

**Table 1:** Maternal and offspring characteristics of the cohort by mode of delivery, N = 11,001.

	Vaginal delivery N = 10234	Cesarean section N = 767	p-value
**Maternal and offspring adiposity**			
Maternal pre-pregnancy BMI, kg/height^2^	21.8±2.9	22.8±3.5	<0.0001
Maternal pre-pregnancy overweight/obesity*,%	12.5	19.8	<0.0001
Offspring BMI at age 17, kg/height^2^	21.4±3.3	21.89±3.5	<0.0001
Offspring overweight/obesity*,%	12.3	16.6	0.001
**Socio-demographic characteristics**			
Maternal Education at birth, %			
0–9 years	22.7	25.4	0.24
10–12 years	40.0	38.7	
13+ years	37.3	35.9	
Socioeconomic Status at birth,%			
Low	22.2	24.4	0.03
Middle	39.3	41.9	
High	38.5	33.8	
Ethnicity,%			
Israel	14.0	14.2	0.63
Asia	28.4	26.4	
Africa	22.6	24.0	
Europe/America	34.9	35.4	
Offspring sex,%			
Female	38.4	38.6	0.92
**Proxies for indication for Cesarean section**			
Toxemia,%	1.2	5.0	<0.0001
Gestational diabetes,%	0.1	0.8	<0.0001
Singletons,%	97.7	93.4	<0.0001
Number of previous pregnancies,%			
0	37.7	37.9	0.99
1	28.9	28.9	
2	18.5	18.4	
≥3	14.9	14.7	
Mother’s age at delivery, years	27.2±5.1	28.9±5.5	<0.0001
Maternal smoking during pregnancy,%	21.3	23.5	0.17
Preterm delivery (≤37 weeks),%	7.2	13.5	<0.0001
Post-term delivery (>41 weeks),%	20.7	24.2	0.023
Birth weight, kg	3.27±0.5	3.18±0.62	<0.0001
High birth weight (>4000 gram),%	5.0	7.3	0.004
Low birth weight (≤2500 gram),%	5.3	11.9	<0.0001
Previous cesarean section,%	1.5	34.8	<0.0001

*Overweight/obesity defined as BMI≥25kg/m^2^

**Fig 1:**
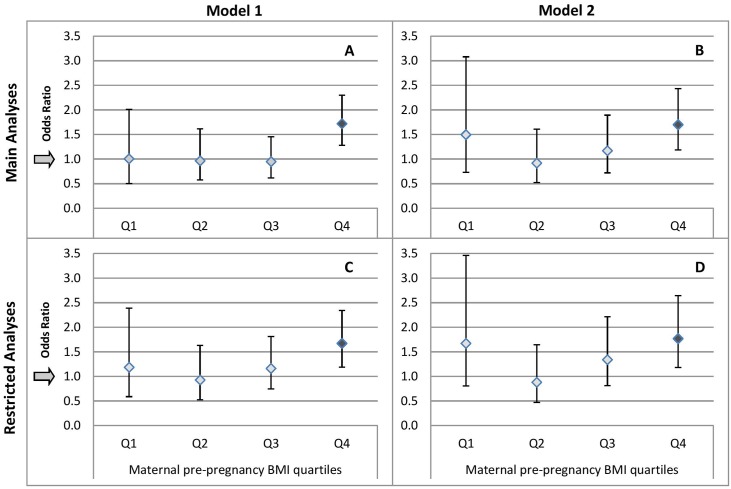
Adjusted odds ratios for overweight and obesity (BMI≥25kg/m^2^) in 17-year-old offspring associated with cesarean vs. vaginal delivery by quartiles of maternal pre-pregnancy BMI. Diamond indicates odds ratio (OR), vertical lines indicate 95% CIs and arrows indicate OR reference of 1. Significant ORs are marked with a dark diamond. Top two graphs (A,B) present results of main analyses and bottom two (C,D) present results of subgroup analyses restricted to singleton first births within the cohort, without toxemia or diabetes in pregnancy. Model 1 (A,C) reflect multivariable models adjusted for maternal education, socioeconomic status, ethnicity and offspring sex. Model 2 (B,D) is further adjusted for proxies for indication for cesarean section, including toxemia, diabetes in pregnancy, multiple pregnancy, birth order, maternal age at delivery, smoking during pregnancy, preterm and post-term delivery, low and high birth weight and previous c-section.

C-Section was associated with offspring overweight/obesity, with adjusted OR of 1.44 (95%CI:1.14–1.82, Table 2; Fig 1, Graphs A,B). Restricting the analyses to singleton first births and excluding pregnancies complicated with toxemia and gestational diabetes yielded similar findings (OR = 1.49,95%CI:1.16–1.92; Fig 1, Graphs C,D).

**Table 2:** Odds Ratio for overweight/obesity (BMI≥25kg/m^2^) in 17-year-old offspring associated with cesarean vs. vaginal delivery.

	Model 1^a^				Model 2^b^			
	OR	p-value	95% CI	p__interaction_^c^	OR	p-value	95% CI	p__interaction_^c^
**Main analyses**	1.42	0.001	1.16–1.74	0.013	1.44	0.002	1.14–1.82	0.014
**Restricted analyses**^d^	1.43	0.002	1.14–1.78	0.106	1.49	0.002	1.16–1.92	0.086

^a^ Adjusted for maternal education, socioeconomic status, ethnicity and offspring sex

^b^ Further adjusted for proxies for indication for cesarean section (c-section), including toxemia, diabetes in pregnancy, multiple pregnancy, birth order, maternal age at delivery, smoking during pregnancy, preterm and post-term delivery, low and high birth weight and previous c-section

^c^ Interaction was examined by adding cross-product terms between upper quartile of maternal pre-pregnancy BMI (vs. rest) and mode of delivery to the multivariable model

^d^ Subgroup of singleton first births within the cohort, without toxemia or diabetes in pregnancy. The restricted sample included 9,160 offspring born vaginally and 631 born by c-section, of which 1,089 and 102 were overweight/obese, respectively.

Significant interaction of ppBMI with mode of delivery was observed (*P interaction* = 0.01), such that the associations of C-Section with overweight/obesity were limited to the upper quartile of ppBMI (OR = 1.70,95%CI:1.18–2.43; Fig 1, Graph B). Additional adjustment for continuous ppBMI, either within each ppBMI-quartile stratum or in models including a multiplicative interaction term, yielded similar results. No significant interaction was observed for BW with C-Section.

## Discussion

In this population-based cohort of individuals born prior to the obesity epidemic, C-Section was associated with a 44% increased odds of offspring overweight/obesity at age 17. Our study further demonstrates that maternal adiposity modifies this association, with a significant C-Section—overweight/obesity association evident only among those born to mothers with the highest pre-pregnancy BMI. C-Section born offspring of mothers in the upper quartile of pre-pregnancy BMI, had 70% increased odds to become overweight/obese at age 17 compared with offspring delivered vaginally, in the same upper quartile of ppBMI.

Accumulating evidence points to an association between C-Section and offspring obesity. One meta-analysis of these studies reported an increased risk of overweight/obesity (OR = 1.33, 95% CI: 1.19, 1.48) for offspring delivered by C-Section compared with those delivered vaginally [5]. Another meta analysis reported a pooled unadjusted OR of 1.26 (95%CI: 1.16, 1.38), however, lacking patient level data prevented adjustment for confounders [6]. Two additional studies, not included in these meta-analyses, reported higher risk of obesity in children born through C-Section (adjusted ORs between 1.2 and 1.68), however these results were not statistically significant most likely due to small sample size (N varied from 1734 to 2988) [8, 13]

Recent data from the Growing Up Today Study (GUTS), a prospective cohort of >22000 offspring, reported a crude RR of 1.30 for obesity associated with C-Section delivery, and this estimate was attenuated to 1.15 in the multivariable model adjusted also for ppBMI [9]. Our findings provide additional support for the GUTS findings and our estimates are clearly in line with theirs. Of note, the magnitudes of the associations reported in most of the above studies are similar, despite the lack of statistical significance in some of them.

The modifying role of ppBMI in the association between C-Section delivery and offspring obesity was explored for the first time only recently. Tun et al. found that infants born vaginally to overweight or obese mothers were 3 times more likely to become overweight, while cesarean-delivered infants of overweight mothers had a 5-fold risk of becoming overweight [11]. One could hypothesize that offspring of obese mothers, who are themselves prone to obesity, may benefit more from exposure to vaginal microbiome during vaginal delivery. There is evidence that the offspring delivered vaginally have a different gut microbiota compared with the offspring delivered in C-Section [10], [14], and that the gut flora of the infant persists into childhood and predicts overweight [15]. Additionally, there is evidence that gut microbiota is linked with obesity [16]. Multiple mediator path modeling revealed that mode of delivery and infant gut microbiota (Firmicutes species richness, especially of the Lachnospiraceae family) sequentially mediated the association between maternal pre-pregnancy overweight and childhood overweight at ages 1 and 3 years [11]. Our findings support and extend these results by showing that the interaction between maternal pre-pregnancy BMI and mode of delivery indeed affects offspring body size, and that this effect is retained beyond early childhood.

Our study subjects represents a population that predated the obesity epidemic. Although it may limit the generalizability of our results, we believe that this is actually a strength of the study, since it demonstrates that the association between C-Section and offspring obesity is consistent across decades. At the time this birth cohort was established, more than four decades ago, obesity rates, as well as rates of C-Section deliveries were significantly lower than current rates. Approximately 13% of the mothers in our cohort had a BMI >25, whereas a recent global report estimates that over than 50% of women >20 years of age in Israel are overweight [17]. Additionally, the rates of gestational diabetes were low in our cohort. As routine screening for gestational diabetes were not available in Israel during the study period, reported rates likely represented the most severe cases. It has been argued that the root of the obesity epidemic is the ubiquitous access to convenient and inexpensive foods with high amounts of salt, sugar, fat, and flavor additives, which drive increased consumption [18]. However, our birth cohort represents a population that was only minimally exposed to western foods. Therefore, the mechanisms underlying this association may be more fundamental, and not necessarily related to the recent obesity epidemic.

Another strength of our study is the availability of reliable measurements of BMI at age 17, through documentation upon military recruitment. These data provided a unique opportunity to follow up offspring into adulthood and avoid reporting bias.

The major limitation of our study is the lack of information on breastfeeding and antimicrobial use which may be associated with changes in the gut microbiota. We also did not have information regarding the indications for C-Section delivery and the timing of membrane rupture, yet aimed to address this limitation by adjusting for proxies for C-Section indication, such as gestational diabetes, toxemia and multiple pregnancy. Finally, lifestyle variables such as physical activity and diet were also unavailable.

In conclusion, our findings suggest that the adverse association between C-Section and offspring adiposity is most pronounced in women whose pre-pregnancy body size is at the upper end of the distribution. In light of the growing rates of obesity in women of reproductive age, as well as the rise in C-Section utilization, more common among obese women [19] these results should be considered in patient-doctor shared decision making when considering C-section without a clear medical indication (e.g. upon maternal request).

## Supporting information

S1 Dataset Dataset plos1.xls. (XLS)

## Abbreviations

BMI: body mass index
BW: birth weight
C-Section: Caesarean Section
JPS: Jerusalem Perinatal Study
ppBMI: maternal pre-pregnancy BMI
